# The Roles of ROS and Caspases in TRAIL-Induced Apoptosis and Necroptosis in Human Pancreatic Cancer Cells

**DOI:** 10.1371/journal.pone.0127386

**Published:** 2015-05-22

**Authors:** Min Zhang, Nanae Harashima, Tamami Moritani, Weidong Huang, Mamoru Harada

**Affiliations:** 1 Department of Biochemistry and Molecular Biology, Ningxia Medical University, Shengli Street, Yinchuan, China; 2 Department of Immunology, Shimane University Faculty of Medicine, Izumi, Shimane, Japan; University of South Alabama Mitchell Cancer Institute, UNITED STATES

## Abstract

Death signaling provided by tumor necrosis factor (TNF)-related apoptosis-inducing ligand (TRAIL) can induce death in cancer cells with little cytotoxicity to normal cells; this cell death has been thought to involve caspase-dependent apoptosis. Reactive oxygen species (ROS) are also mediators that induce cell death, but their roles in TRAIL-induced apoptosis have not been elucidated fully. In the current study, we investigated ROS and caspases in human pancreatic cancer cells undergoing two different types of TRAIL-induced cell death, apoptosis and necroptosis. TRAIL treatment increased ROS in two TRAIL-sensitive pancreatic cancer cell lines, MiaPaCa-2 and BxPC-3, but ROS were involved in TRAIL-induced apoptosis only in MiaPaCa-2 cells. Unexpectedly, inhibition of ROS by either *N*-acetyl-L-cysteine (NAC), a peroxide inhibitor, or Tempol, a superoxide inhibitor, increased the annexin V-/propidium iodide (PI)^+^ early necrotic population in TRAIL-treated cells. Additionally, both necrostatin-1, an inhibitor of receptor-interacting protein kinase 1 (RIP1), and siRNA-mediated knockdown of RIP3 decreased the annexin V^-^/PI^+^ early necrotic population after TRAIL treatment. Furthermore, an increase in early apoptosis was induced in TRAIL-treated cancer cells under inhibition of either caspase-2 or -9. Caspase-2 worked upstream of caspase-9, and no crosstalk was observed between ROS and caspase-2/-9 in TRAIL-treated cells. Together, these results indicate that ROS contribute to TRAIL-induced apoptosis in MiaPaCa-2 cells, and that ROS play an inhibitory role in TRAIL-induced necroptosis of MiaPaCa-2 and BxPC-3 cells, with caspase-2 and -9 playing regulatory roles in this process.

## Introduction

Members of the tumor necrosis factor (TNF) cytokine family, such as TNFα and Fas ligand (FasL), play important roles in inflammation and immunity [1]. Although TNF-related apoptosis-inducing ligand (TRAIL) is a member of this family, this molecule can induce cancer cell death while causing almost no cytotoxicity to normal cells [2]. There are positive and negative receptors; death receptor (DR)4 and DR5 provide pro-apoptotic signaling, whereas decoy receptor (DcR)1 and DcR2 inhibit apoptotic signaling [3]. Normal cells are reported to show TRAIL-resistance with their preferential expression of DcRs [4]. Therefore, TRAIL and its DR are expected to be useful anti-cancer molecules and targets and have been used and targeted in several clinical trials [5, 6]. Binding of TRAIL to DR on cancer cells provides caspase-8-dependent death signaling and triggers the ‘extrinsic’ apoptotic pathway [7]. Activation of caspase-8 transforms Bid to tBid, thereby promoting the mitochondria-mediated caspase-9-dependent ‘intrinsic’ apoptotic pathway [8]. Alternatively, ROS (reactive oxygen species) also play important roles in cell death and signaling [9]. ROS induce intrinsic apoptosis by triggering DNA damage [10]. Conversely, DNA damage induces ROS production [11]. DNA damage and/or ROS production can trigger caspase-9-dependent apoptosis. Additionally, some reports suggested that ROS is involved in apoptosis in TRAIL-treated human cancer cells [12–14]. However, the roles of ROS in TRAIL-induced cancer cell death have yet to be investigated fully.

In addition to apoptosis, necroptosis is now recognized as another form of programmed cell death [15, 16]. Necroptosis is programmed necrosis that can be activated upon stimulation by TNFα, FasL, or TRAIL. The roles and mechanisms of apoptosis and necrosis have been well established, whereas those of necroptosis have been the focus of recent investigations [16–18]. Necroptosis has received much attention as a type of cell death that induces inflammation [17]. Under caspase-8 inhibition, receptor-interacting protein kinase 1 (RIP1) and RIP3 form a complex and trigger necroptosis [19, 20]; additional recent reports have revealed that RIP3 plays a central role in the process [18, 21]. Although some reports suggest that TRAIL can induce necroptosis in cancer cells [22–24], the mechanisms have not been elucidated fully.

This study investigated the roles of ROS and caspases in TRAIL-induced apoptosis and necroptosis of human pancreatic cancer cells. Among four human pancreatic cancer cell lines, ROS levels were elevated in only two TRAIL-sensitive lines: MiaPaCa-2 and BxPC-3. However, ROS played a pro-apoptotic role only in MiaPaCa-2 cells, but not in BxPC-3 cells. In these experiments, we found that TRAIL treatment under ROS inhibition increased the population of annexin V^-^/propidium iodide (PI)^+^ early necrotic cells in MiaPaCa-2 and BxPC-3 cells, suggesting that ROS played an inhibitory role in TRAIL-induced necroptosis in both cell lines. In addition, necrostatin-1 (a RIP1 inhibitor) decreased the annexin V^-^/PI^+^ population in these TRAIL-treated cells, and siRNA-mediated knockdown of RIP3 showed similar results in BxPC-3 cells, implying that the cell death observed was caused by necroptosis. Finally, necroptosis was promoted by TRAIL treatment under the inhibition of either caspase-9 or -2, with the latter considered an ‘orphan’ caspase whose roles in cell death have not been elucidated fully [25, 26]. Together, these findings indicate that ROS are increased in TRAIL-sensitive MiaPaCa-2 and BxPC-3 cells, but that ROS are involved in apoptosis only in TRAIL-treated MiaPaCa-2 cells. Our findings also showed that ROS and caspase-9/-2 play regulatory roles in the TRAIL-induced necroptosis of human pancreatic cancer cells.

## Materials and Methods

### Cell lines

Two human pancreatic cancer cell lines (AsPC-1 and BxPC-3) were purchased from the American Type Culture Collection (Manassas, VA, USA). Two other human pancreatic cancer cell lines (MiaPaCa-2 and Panc-1) were kindly provided by Dr. K. Takenaga (Shimane University Faculty of Medicine) [27]. These cell lines were maintained in DMEM (Sigma-Aldrich, St. Louis, MO, USA) supplemented with 10% fetal calf serum (Invitrogen, Grand Island, NY, USA) and 20 μg/ml gentamicin (Sigma-Aldrich). PrEC is a normal prostate epithelial cell line purchased from Lonza (Walkersville, MD, USA) and was maintained in PrEBM (Lonza).

### Cell viability assay

Cell viability was analyzed using the WST-8 assay (Nacalai Tesque, Kyoto, Japan). At the end of the incubation period, 10 μl WST-8 solution was added to each well, and the plates were incubated for an additional 3 h. Absorbance in each well was measured at 560 nm using a microplate reader (Beckman Coulter, Brea, CA, USA).

### Reagents

For inhibition assays, the following inhibitors were added 1 h before the addition of TRAIL: pan-caspase inhibitor Z-VAD-FMK (Enzo Life Sciences, Farmingdale, NY, USA), caspase-8 inhibitor Z-IETD-FMK (R&D Systems, Minneapolis, MN, USA), caspase-9 inhibitor Z-LEHD-FMK (R&D Systems), and caspase-2 inhibitor Z-VDVAD-FMK (R&D Systems). *N*-acetyl-l-cysteine (NAC) was purchased from Nacalai Tesque. Tempol and necrostatin-1 were purchased from Santa Cruz Biotechnology, Santa Cruz, CA, USA.

### Detection of DR and decoy receptor (DcR) expression on cells

To examine the expression of DR4 (CD261) and DR5 (CD262), cells were incubated with either anti-DR4 (eBioscience, San Diego, CA, USA) or anti-DR5 (eBioscience), followed by staining with FITC-conjugated goat anti-mouse IgG (H+L) (KPL, Gaithersburg, MD, USA). To examine the expression of DcR1 (CD263) and DcR2 (CD264), cells were stained with either FITC-conjugated anti-DcR1 (CD263) (GeneTex, Irvine, CA, USA), or FITC-conjugated anti-DcR2 (CD264) (GeneTex). For these incubations, isotype-matched FITC-conjugated mouse IgG1was used as a control. Analysis was performed using a FACSCalibur flow cytometer (Becton Dickinson, Franklin Lakes, NJ, USA).

### Apoptosis assay

Cell death was assessed using the Annexin V-FITC Apoptosis Detection Kit (BioVision, Mountain View, CA, USA) and PI. Each caspase inhibitor (20 μM), or the same volume of DMSO as a vehicle control, was added 1 h before the addition of TRAIL. To examine effects of NAC and Tempol on TRAIL-induced apoptosis, cells were cultured with TRAIL (50 ng/mL) with or without NAC (10 mM) or Tempol (1 mM) for 24 h. To examine effects of necrostatin-1, necrostatin-1 (20 μM) was added at the initiation of culture. After staining with annexin V-FITC/PI, flow cytometric analysis was performed. Analysis was performed using a FACSCalibur flow cytometer.

### ROS measurement

Intracellular ROS were measured using carboxy-H_2_DCFDA (Molecular Probes, Carlsbad, CA, USA). Cells were cultured with TRAIL (50 ng/mL). After 6 h for MiaPaCa-2 and 12 h for the other lines, carboxy-H_2_DCFDA (50 μM) was added and cultured additionally for 30 min. Collected cells were analyzed by flow cytometry. Each caspase inhibitor (20 μM) was added 1 h before the addition of TRAIL.

### Immunoblotting

Cells were lysed with a mammalian protein extraction reagent (M-PER; Thermo Scientific, Rockford, IL, USA) containing a protease-inhibitor cocktail (Nacalai Tesque). Equal amounts of protein were resolved on 4–12% gradient or 12% SDS-PAGE gels and transferred to polyvinylidene fluoride membranes. The membranes were blocked and the blots incubated with the following primary antibodies: anti-RIP3 (13526; Cell Signaling Technology, Danvers, MA, USA), anti-caspase-3 (9668; Cell Signaling Technology), anti-caspase-8 (M032-3; Medical and Biological Laboratories, Nagoya, Japan), anti-caspase-9 (9508; Cell Signaling Technology), anti-caspase-2 (2224; Cell Signaling Technology), anti-β-actin (BioLegend, San Diego, CA, USA), or anti-α-tubulin (Santa Cruz Biotechnology). After washing, room temperature incubation of membranes for 30 min with either goat anti-rabbit or goat anti-mouse alkaline phosphatase-conjugated secondary antibodies (Invitrogen) was used to detect the primary antibodies. Protein bands were visualized using CDP-star chemiluminescence and imaged using an ImageQuant LAS-4000 system (FujiFilm, Tokyo, Japan).

### Transfection of small interfering RNA (siRNA)

Transfection of siRNA was performed using Lipofectamine RNAiMAX (Invitrogen), according to the manufacturer’s instructions. RIP3 siRNA (sc-61482) was purchased from Santa Cruz Biotechnology. The control siRNA (6568) was purchased from Cell Signaling Technology. Three days after siRNA transfection, the cells were used for subsequent experiments.

### Statistical analyses

Data were evaluated statistically using unpaired two-tailed Student’s *t*-tests. A *P* value of less than 0.05 was considered to indicate statistical significance.

## Results

### Production of ROS in TRAIL-sensitive pancreatic cancer cells

**Fig 1:**
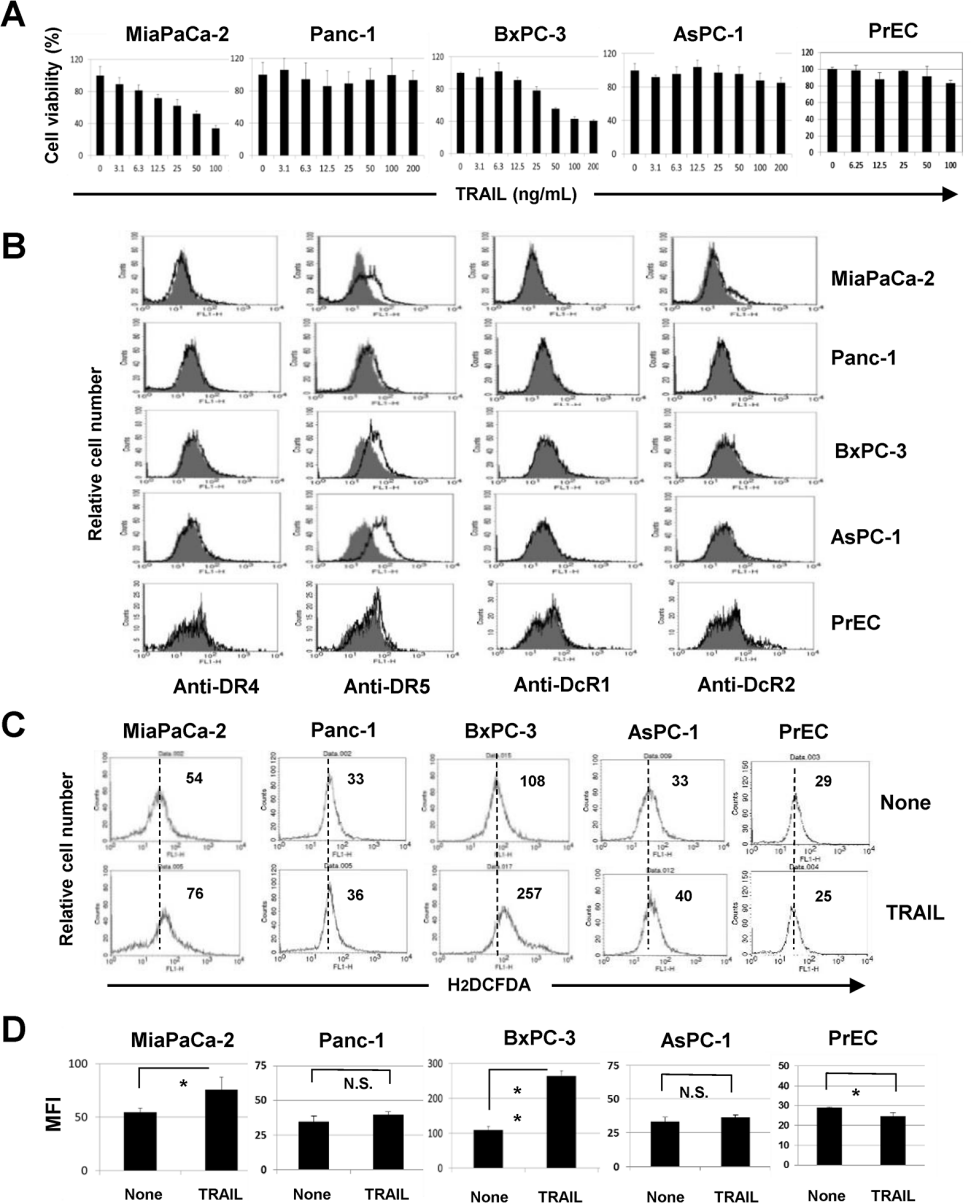
ROS production in TRAIL-sensitive human pancreatic cancer cell lines. (A) Four human pancreatic cancer lines and PrEC cells were cultured in the presence of TRAIL. After 48 h, cell viability was determined by the WST-8 assay. The data shown represent the mean of three wells. (B) The expression of DR4, DR5, DcR1, and DcR2 in five cell lines was examined by flow cytometry. The line represents staining with mAb specific to either DR4 or DR5, followed by a FITC-conjugated secondary antibody. Solid gray represents staining with FITC-conjugated anti-mouse IgG alone. Regarding the expression of DcR1 and DcR2, the line represents staining with mAb specific to DcR1 and DcR2; solid gray represents staining with isotype-matched FITC-conjugated anti-mouse IgG. (C) Five cell lines were cultured with TRAIL (50 ng/mL). After 6 h for MiaPaCa-2 and 12 h for the other four lines, these cells were cultured with carboxy-H_2_DCFDA (50 μM) for 30 min and examined for their ROS levels by flow cytometry. The number represents the mean fluorescence intensity. (D) The data shown represent the mean of three wells. MFI: mean fluorescence intensity. *P<0.05, **P<0.01, N.S., not significant.

We first examined the sensitivity of four human pancreatic cancer cell lines and a normal prostate epithelial cell line PrEC to TRAIL treatment. We selected these cancer cell lines because they have been well-characterized for their mutations in *K-ras* and *p53* [28] and because we previously examined their TRAIL sensitivity [29]. As a result, the viability of both MiaPaCa-2 and BxPC-3 cells decreased in the presence of TRAIL in a dose-dependent manner, whereas the other three lines (Panc-1, AsPC-1, and PrEC) showed no clear sensitivity toward TRAIL (Fig 1A). We next examined the expression of TRAIL receptors on these cells (Fig 1B). The expression of DR4 was almost undetectable in all cell lines. Although DR5 expression on Panc-1 and PrEC was low, all cell lines were positive for DR5. In terms of decoy receptors, MiaPaCa-2 and PrEC cells were partially positive for DcR2. We next determined whether ROS were produced in these cell lines and found that TRAIL treatment significantly increased ROS levels only in MiaPaCa-2 and BxPC-3 cells (*P*<0.05 for MiaPaCa-2, *P*<0.01 for BxPC-3) (Fig 1C and 1D). PrEC cells reduced the level of ROS after TRAIL treatment. These results indicated that ROS are produced only in TRAIL-sensitive pancreatic cancer cell lines (MiaPaCa-2 and BxPC-3).

### Inhibition of ROS decreased TRAIL-induced apoptosis only in MiaPaCa-2 cells

**Fig 2:**
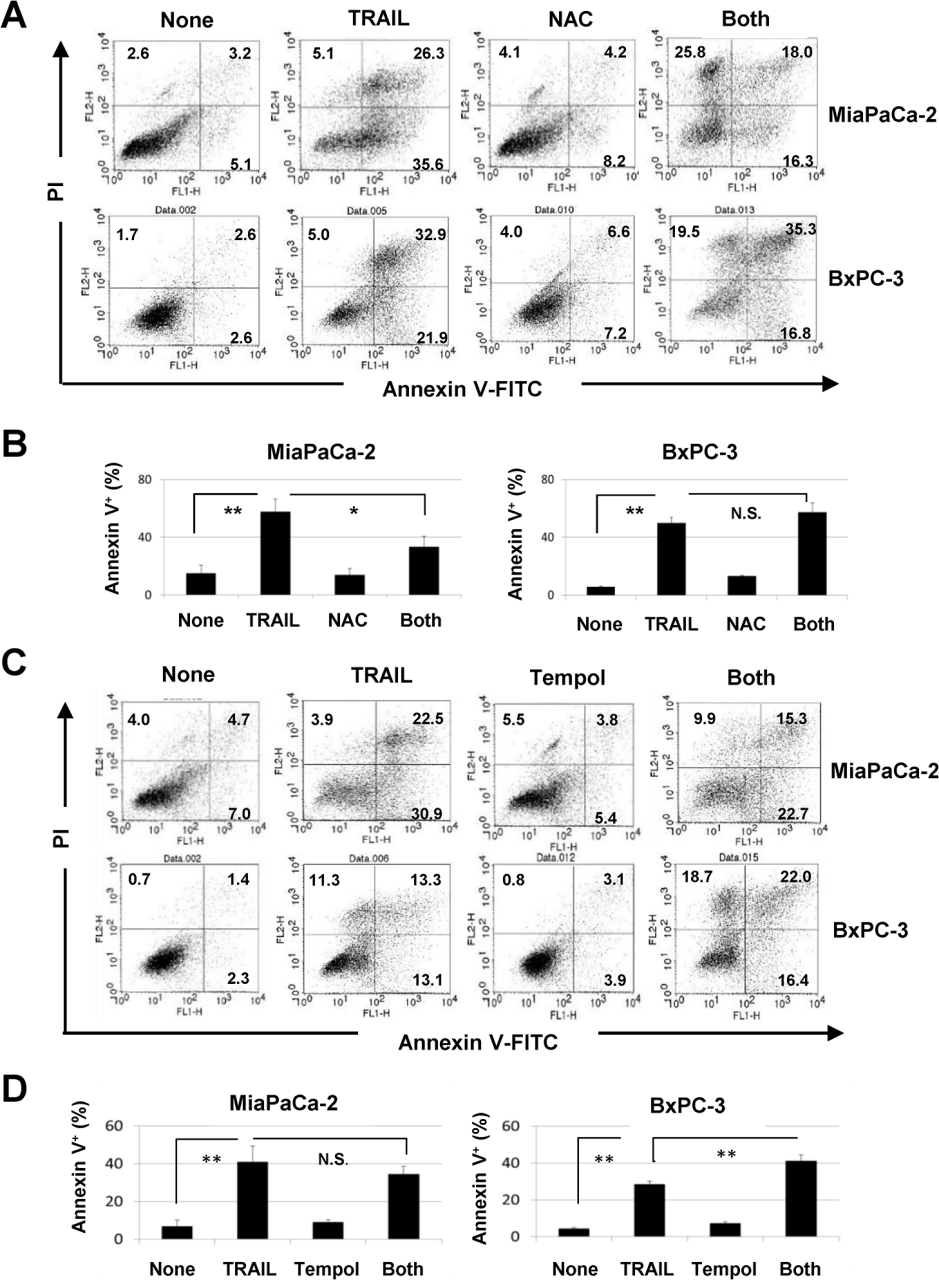
ROS-dependent apoptosis in TRAIL-treated MiaPaCa-2 cells. (A) MiaPaCa-2 and BxPC-3 cells were cultured with TRAIL (50 ng/mL) with or without NAC (10 mM) for 24 h. After staining with annexin V-FITC/PI, flow cytometric analysis was performed. The numbers represent the proportions of each subset. (B) The percentages of annexin V^+^ cells are shown. (C) Both cell lines were cultured with TRAIL (50 ng/mL) with or without Tempol (1 mM) for 24 h and analyzed by flow cytometry. (D) The percentages of annexin V^+^ cells are shown. All data points shown represent the mean of three culture wells. *P<0.05, **P<0.01, N.S., not significant.

We next examined the effects of two ROS inhibitors, NAC and Tempol [30], on TRAIL-induced apoptosis of TRAIL-sensitive MiaPaCa-2 and BxPC-3 cells. TRAIL significantly increased the percentages of annexin V^+^ cells among both cell lines (*P*<0.01). The addition of NAC, a peroxide inhibitor, significantly decreased the percentage of annexin V^+^ TRAIL-treated MiaPaCa-2 cells (*P*<0.05) but failed to decrease apoptosis in TRAIL-treated BxPC-3 cells (Fig 2A and 2B). Alternatively, the addition of Tempol, a superoxide inhibitor, had no effect on TRAIL-induced apoptosis of MiaPaCa-2 cells but increased it in TRAIL-treated BxPC-3 cells (*P*<0.01) (Fig 2C and 2D). These results indicate that ROS, peroxide and superoxide, exert opposite effects on the two TRAIL-sensitive cell lines; peroxide plays a pro-apoptotic role in TRAIL-treated MiaPaCa-2 cells, but superoxide is anti-apoptotic in TRAIL-treated BxPC-3 cells.

### RIP1- and RIP3-dependent necroptosis in TRAIL-treated pancreatic cancer cells under ROS inhibition

**Fig 3:**
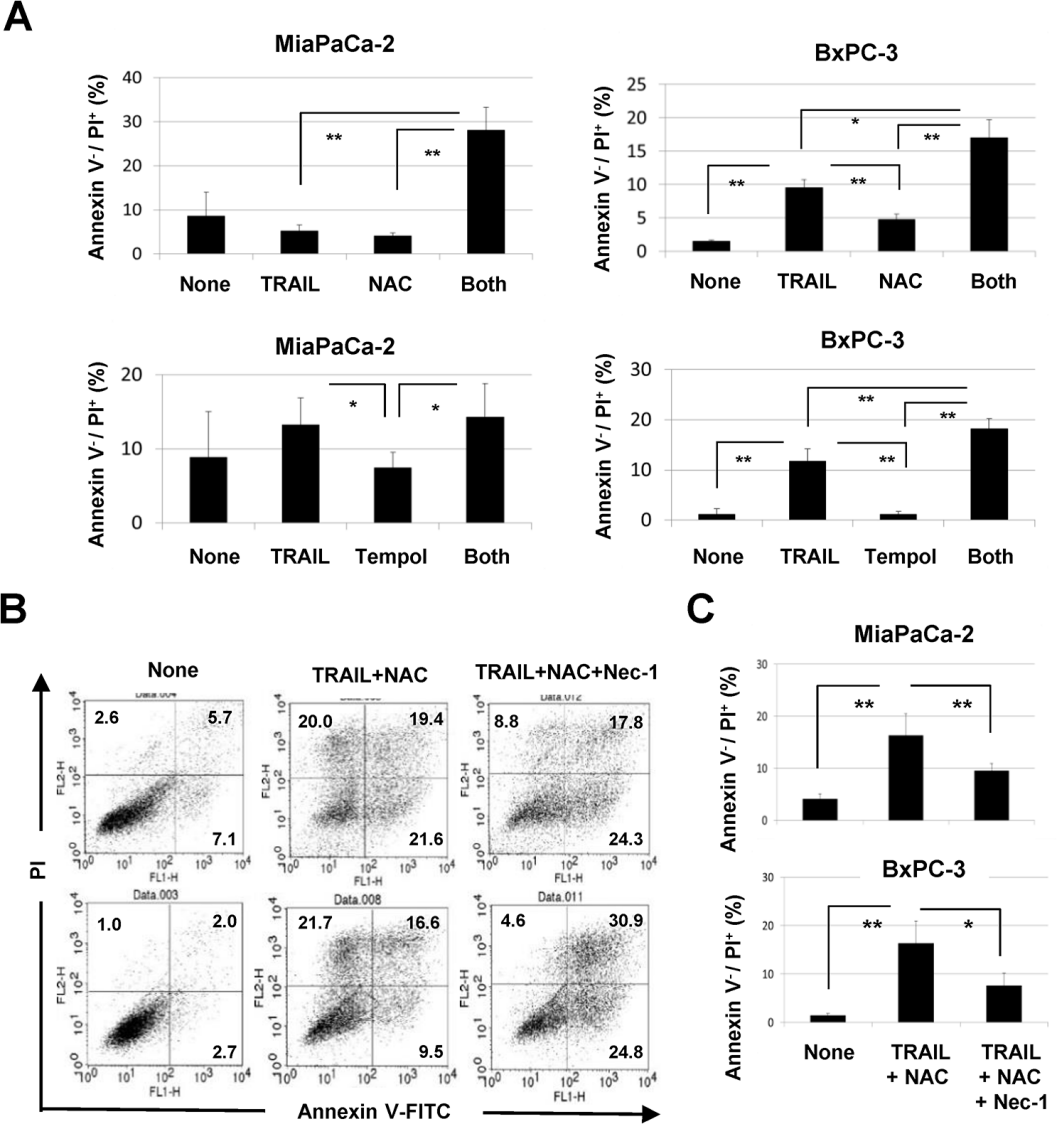
TRAIL-induced necroptosis in human pancreatic cancer cells under ROS inhibition. (A) The percentages of annexin V^-^/PI^+^ cells were calculated based on the results of Fig 2. (B) Both cell lines were cultured with TRAIL (50 ng/mL) and NAC (10 mM), with or without necrostatin-1 (20 μM) for 24 h. After staining with annexin V-FITC/PI, flow cytometric analysis was performed. The numbers represent the proportions of each subset. (C) The percentages of annexin V^-^/PI^+^ cells were calculated. All data points shown represent the mean of three culture wells. *P<0.05, **P<0.01.

During examination of the effects of ROS inhibition on TRAIL-induced apoptosis of MiaPaCa-2 and BxPC-3 cells, we found that the percentages of annexin V^-^/PI^+^ early necrotic cells were increased by TRAIL treatment under ROS inhibition (Fig 2A and 2C). The results of annexin V^-^/PI^+^ early necrotic cells are calculated and presented in Fig 3A. The addition of NAC significantly increased the percentages of annexin V^-^/PI^+^ TRAIL-treated cells (*P*<0.01 for MiaPaCa-2, *P*<0.05 for BxPC-3). A similar result was observed when Tempol was added in TRAIL-treated BxPC-3 cells (*P*<0.01 for BxPC-3), but not in MiaPaCa-2 cells. Since annexin V^-^/PI^+^ cells represent early necrotic cells, these results suggest that TRAIL induced programmed necrosis (necroptosis) under ROS inhibition, especially peroxide inhibition. We next asked whether the addition of necrostatin-1, an inhibitor of RIP1 and of necrosis [31], could decrease these early necrotic cells. As shown in Fig 3B and 3C, the combination of TRAIL and NAC significantly increased the proportions of annexin V^-^/PI^+^ MiaPaCa-2 and BxPC-3 cells (*P*<0.01 for MiaPaCa-2 and BxPC-3), whereas the addition of necrostatin-1 significantly decreased them (*P*<0.01 for MiaPaCa-2, *P*<0.05 for BxPC-3).

**Fig 4:**
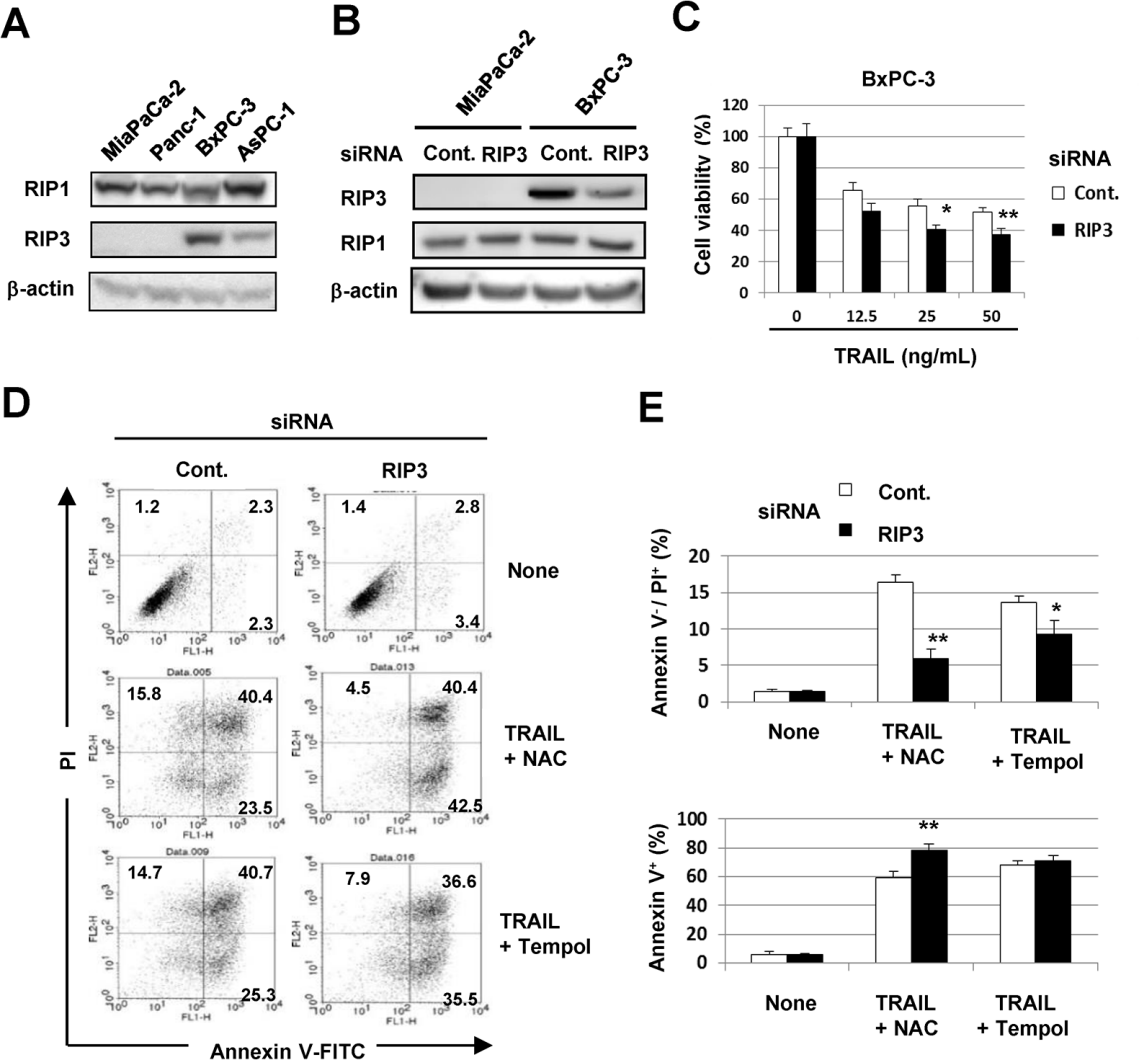
RIP3-dependent necroptosis in TRAIL-treated BxPC-3 cells under ROS inhibition. (A) The expression of RIP3 and RIP1 protein was examined in four cancer cell lines. β-Actin was used as a control. (B) MiaPaCa-2 and BxPC-3 cells were transfected with control siRNA or RIP3 siRNA. Three days after transfection, the cells were harvested and examined for RIP3 and RIP1 protein expression. (C) BxPC-3 cells transfected with either control siRNA or RIP3 siRNA 3 days prior were cultured with TRAIL. After 48 h, cell viability was determined by the WST-8 assay. (D) BxPC-3 cells transfected with either control siRNA or RIP3 siRNA 3 days prior were cultured with TRAIL and with either NAC (10 mM) or Tempol (1 mM) for 24 h. After staining with annexin V-FITC/PI, flow cytometric analysis was performed. The numbers represent the proportions of each subset. (E) The percentages of annexin V^-^/PI^+^ cells and annexin V^+^ cells were calculated. All data points shown represent the mean of three culture wells. *P<0.05, **P<0.01.

RIP1 and RIP3 are critical molecules for necroptosis [18–21]. Therefore, we examined the expression of RIP1 and RIP3 in four pancreatic cancer cell lines and found that all were positive for RIP1, while MiaPaCa-2 and Panc-1 were negative for RIP3 (Fig 4A). We further examined the effect of siRNA-mediated knockdown of RIP3 on necroptosis induced by coincubation with TRAIL and NAC. Transfection of RIP3 siRNA decreased the RIP3 protein expression in BxPC-3 cells, but showed no effect on the expression of RIP1 (Fig 4B). Knockdown of RIP3 slightly, but significantly, decreased the cell viability in response to TRAIL (*P*<0.05 at 25 ng/mL, *P*<0.01 at 50 ng/mL) (Fig 4C). As shown in Fig 4D and 4E, selective knockdown of RIP3 significantly decreased the proportions of annexin V^-^/PI^+^ cells after TRAIL treatment of BxPC-3 cells cultured with either NAC or Tempol (*P*<0.01 for NAC, *P*<0.05 for Tempol). Interestingly, inhibition of peroxide by NAC significantly increased the proportion of annexin V^+^ apoptotic cells in RIP3 siRNA-transfected and TRAIL-treated BxPC-3 cells (*P*<0.01). These results suggested that RIP1-dependent necroptosis is promoted in TRAIL-treated MiaPaCa-2 and BxPC-3 cells under inhibition of peroxide, and that TRAIL treatment under inhibition of ROS (peroxide and superoxide) promotes RIP3-dependent necroptosis in BxPC-3 cells.

### The roles of caspases in TRAIL-induced apoptosis and necroptosis

**Fig 5:**
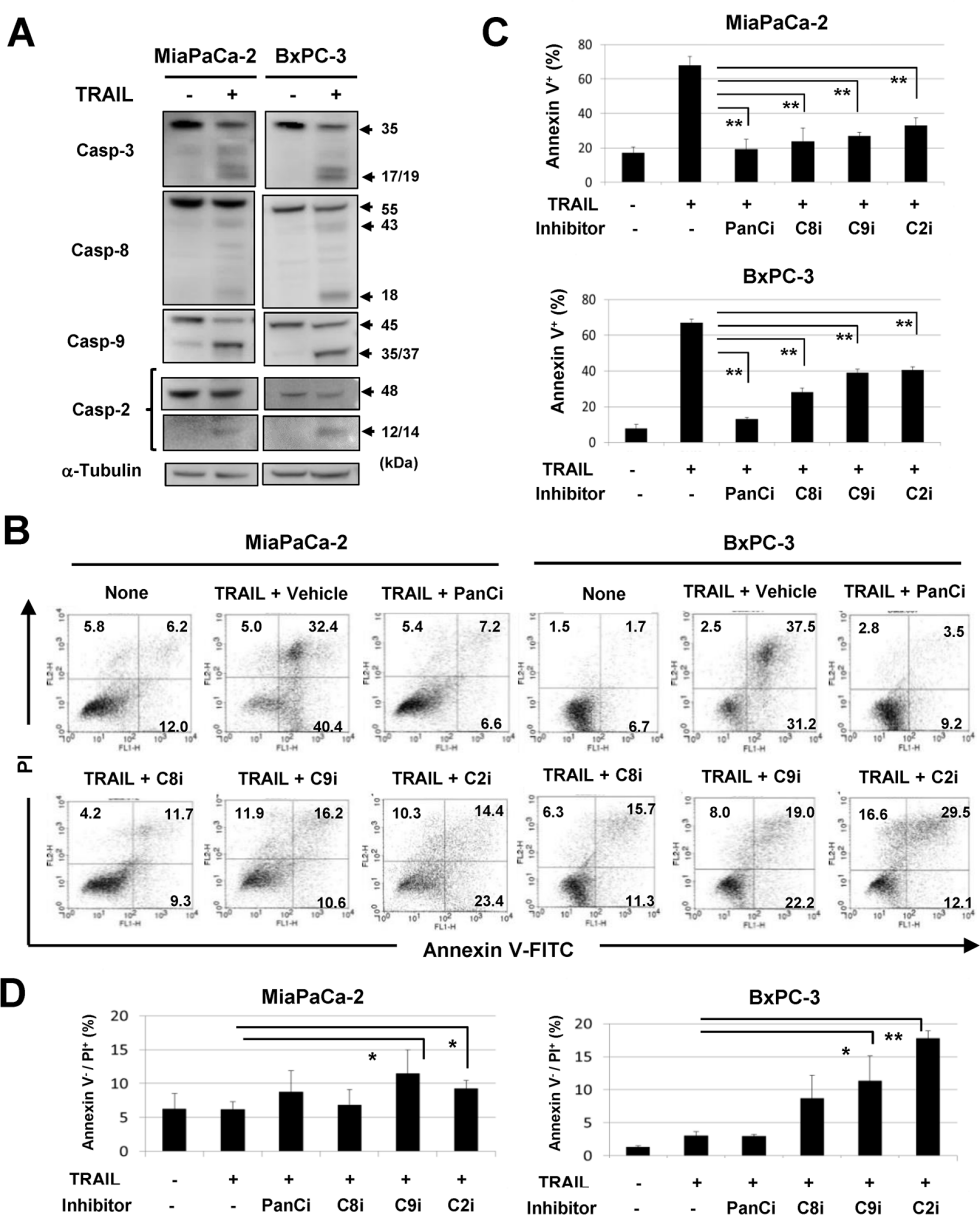
The roles of caspases in apoptosis and necroptosis of TRAIL-treated cells. (A) MiaPaCa-2 and BxPC-3 cells were cultured with TRAIL (50 ng/mL) for 12 h, and protein expression levels of caspase-3, caspase-8, caspase-9, and caspase-2 were evaluated by immunoblot. α-Tubulin was used as the control. (B) MiaPaCa-2 and BxPC-3 cells were cultured with TRAIL (50 ng/mL) in the presence of a panel of caspase inhibitors (20 μM) for 24 h. After staining with annexin V-FITC/PI, flow cytometric analysis was performed. The numbers represent the proportions of each subset. The percentages of annexin V^+^ cells (C) and annexin V^-^/PI^+^ cells (D) were determined by flow cytometry. All data points shown represent the mean of three culture wells. *P<0.05, **P<0.01. panCi, pan-caspase inhibitor; C9i, caspase-9 inhibitor; C8i, caspase-8 inhibitor; C2i, caspase-2 inhibitor. Vehicle controls received an equal volume of DMSO.

We next examined the roles of caspases in apoptosis and necroptosis in TRAIL-treated MiaPaCa-2 and BxPC-3 cells. TRAIL treatment activated caspase-3, -8, and -9 in both cell lines (Fig 5A). Since the role of caspase-2 in cell death has not been established fully [25, 32], we monitored this caspase and found that TRAIL treatment also activated caspase-2. We further examined the effects of a panel of caspase inhibitors on TRAIL-induced apoptosis (Fig 5B and 5C). The addition of the pan-caspase inhibitor Z-VAD profoundly inhibited TRAIL-induced apoptosis in both cell lines, and inhibitors of caspase-8, -9, or -2 also significantly inhibited TRAIL-induced apoptosis (*P*<0.01). We examined the effect of these inhibitors on annexin V^-^/PI^+^ early apoptotic cells (Fig 5D). Interestingly, inhibitors of either caspase-9 or -2 significantly increased the proportions of early apoptotic cells in TRAIL-treated MiaPaCa-2 and BxPC-3 cultures (*P*<0.05 or *P*<0.01). These results indicate that a panel of caspases participate in TRAIL-induced apoptosis in these lines and suggest that caspase-9 and -2 play an inhibitory role in TRAIL-induced necroptosis.

### No crosstalk between caspase-2/-9 and ROS in TRAIL-treated cancer cells

**Fig 6:**
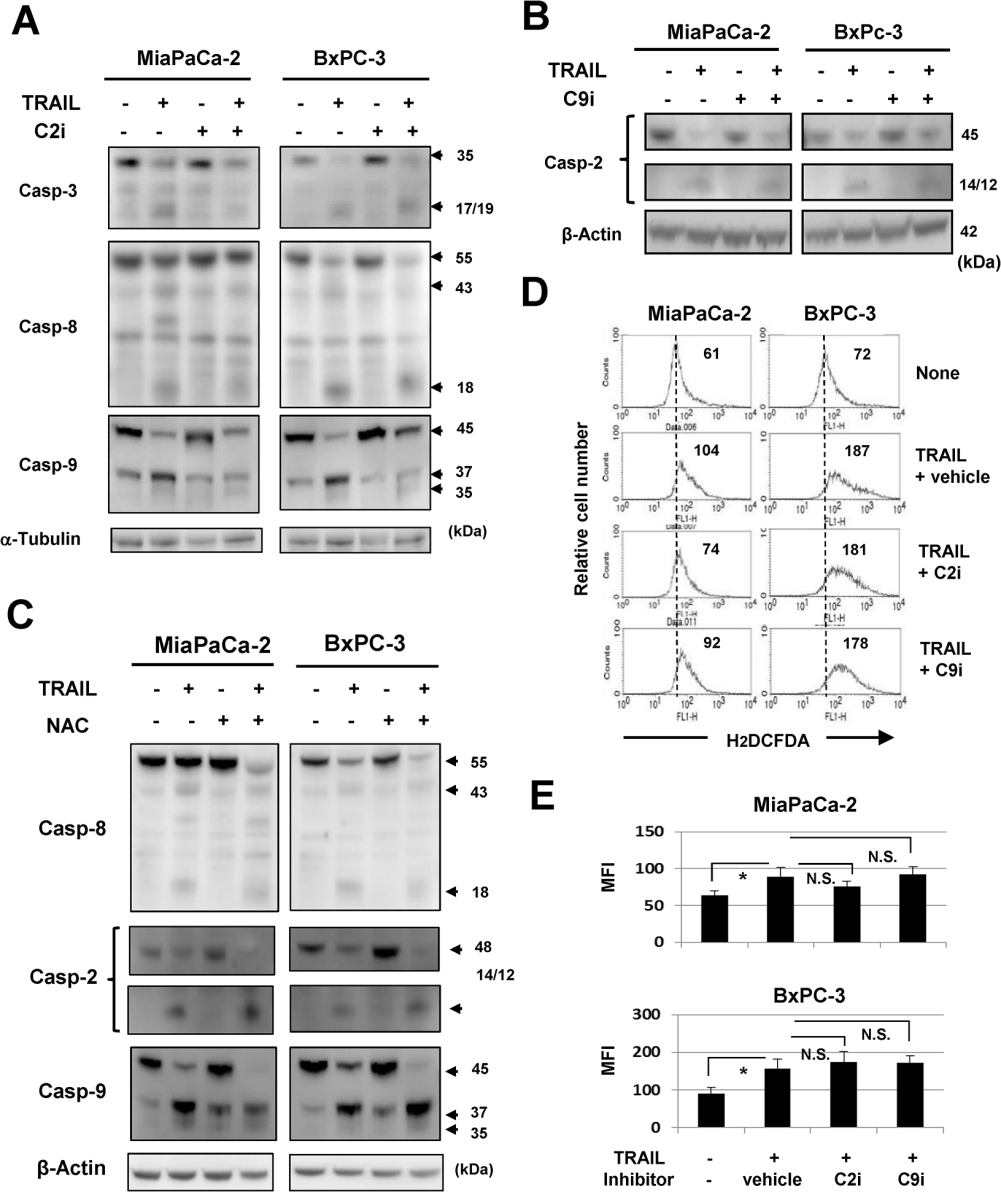
No crosstalk among caspase-2/-9 and ROS in TRAIL-treated cancer cells. (A) MiaPaCa-2 and BxPC-3 cells were cultured with TRAIL (50 ng/mL) with or without caspase-2 inhibitor (20 μM) for 12 h, and the protein expression levels of caspase-3, -8, and -9 were evaluated by immunoblot. α-Tubulin was used as the control. (B) MiaPaCa-2 and BxPC-3 cells were cultured with TRAIL (50 ng/mL) with or without caspase-9 inhibitor (20 μM) for 12 h, and the protein expression of caspase-2 was evaluated by immunoblot. β-Actin was used as the control. (C) MiaPaCa-2 and BxPC-3 cells were cultured with TRAIL (50 ng/mL) with or without NAC (10 mM) for 12 h, and the protein expression of caspase-8, -2, and -9 was evaluated by immunoblot. β-Actin was used as the control. (D) MiaPaCa-2 and BxPC-3 cells were cultured with TRAIL (50 ng/mL) with the indicated inhibitors (20 μM). As a vehicle control, an equal volume of DMSO was added. After 6 h for MiaPaCa-2 and 12 h for BxPC-3, these cells were cultured with carboxy-H_2_DCFDA for 30 min and examined for ROS levels by flow cytometry. The number represents the mean fluorescence intensity. (E) Data represent the mean of three culture wells. MFI: mean fluorescence intensity. *P<0.05, N.S., not significant.

We next examined the relationship between caspase-9, -2, and ROS in TRAIL-treated pancreatic cancer cells. The addition of a caspase-2 inhibitor inhibited TRAIL-induced caspase-9 cleavage in two cell lines but had no apparent suppressive effect on caspase-8 activation (Fig 6A). A caspase-9 inhibitor did not suppress caspase-2 activation (Fig 6B). The addition of NAC failed to suppress TRAIL-induced activation of caspase-9 and -2 (Fig 6C). Finally, we examined the effects of caspase-2 or -9 inhibition on ROS production by TRAIL-treated cells. No clear suppression of ROS production was noted (Fig 6D and 6E). These results indicate that caspase-2 is upstream of caspase-9, and that there is no crosstalk between these caspases and ROS in TRAIL-treated MiaPaCa-2 and BxPC-3 cells.

## Discussion

Since pancreatic cancer is highly resistant to conventional therapies and is associated with a poor prognosis [33], new treatment modalities are required. TRAIL may be useful therapeutically, because this molecule can induce cell death in many types of cancers while causing almost no cytotoxicity to normal cells [2]. In this study, we first investigated the roles of ROS in TRAIL-induced apoptosis of human pancreatic cancer cell lines. During these experiments, we unexpectedly found that inhibition of ROS promoted TRAIL-induced necrosis of cancer cells. We then determined that this cell death was necroptosis.

ROS play diverse roles in many types of cells. Low physiological levels of ROS function as second messengers in intracellular signaling and are required for normal cell functions, whereas excessive ROS impair cell functions and promote cell death [34]. TRAIL has been shown to induce ROS generation in cancer cells [35], and antioxidants block DR signaling-mediated apoptosis [36, 37], suggesting that ROS are mediators of death ligand-induced apoptosis. Here, we found that ROS were produced only in TRAIL-sensitive pancreatic cancer cell lines, and that peroxide contributes, at least partially, to TRAIL-induced apoptosis in MiaPaCa-2 cells (Fig 2B). Alternatively, the inhibitor of superoxide Tempol unexpectedly increased apoptosis in TRAIL-treated BxPC-3 cells (Fig 2D), suggesting that superoxide acts as an anti-apoptotic mediator under these conditions. What was the mechanism of the inhibitory effect of superoxide on apoptosis in TRAIL-treated BxPC-3 cells? ROS, especially superoxide, can induce autophagy [26, 38, 39], and autophagy can function cytoprotectively [40]. We recently reported that autophagy plays an anti-apoptotic role in TRAIL-treated pancreatic cancer cells [29]. Therefore, we explored whether autophagy was induced in BxPC-3 cells and the superoxide inhibitor Tempol suppressed ROS-induced autophagy, resulting in the promotion of apoptosis. We evaluated the level of autophagy in cancer cells by examining the expression of LC3. As a result, autophagy was strongly induced in BxPC-3 cells compared with the other pancreatic cancer cell lines, while Tempol failed to inhibit the expression of LC3-type II, as a marker of autophagy [41], in BxPC-3 cells “S1 Fig”. Autophagy does not contribute to ROS-mediated protection in TRAIL-treated BxPC-3 cells. Further studies are required to elucidate the precise mechanism.

Some ligands, including TNFα, FasL, and TRAIL, can induce both apoptosis and necroptosis [1]. The mechanisms that dictate the cellular decision to undergo apoptosis or necroptosis have been investigated intensively. In apoptosis, caspases are the executioners of apoptosis; however, these proteases have no positive role in necroptosis [15]. ROS are proposed to be executioners of necroptosis [42], and some cell types, such as mouse L929 and embryonic fibroblasts, produce ROS in response to TNFα and show necroptosis [43, 44]. Additionally, treatment with antioxidants inhibits necroptosis in some cell types [44]. However, ROS are not required for necroptosis in all cell types and antioxidants are unable to protect some cell lines from this type of cell death [45, 46]. These lines of evidence suggest that ROS-mediated necroptosis is likely a cell type-dependent phenomenon. In this study, we showed that the addition of NAC to inhibit peroxide promoted necroptosis upon TRAIL stimulation in two human pancreatic cancer cell lines (Fig 3A). This increase was decreased by necrostatin-1, a RIP1 inhibitor [30] (Fig 3B and 3C), and siRNA-mediated RIP3 knockdown decreased necroptosis in TRAIL-treated BxPC-3 cells under inhibition of ROS (peroxide and superoxide) (Fig 4D and 4E). These results indicated that ROS play an inhibitory role in TRAIL-induced necroptosis in BxPC-3 cells. Alternatively, MiaPaCa-2 cells were negative for RIP3 (Fig 4), suggesting that RIP3 was dispensable for necroptosis in TRAIL-treated MiaPaCa-2 cells.

Our results suggest that no obvious crosstalk between ROS and caspase-2/caspase-9 exists in the experimental system. It remains unresolved how an early necrotic population was induced by TRAIL treatment under inhibition of caspase-2 or -9. Inhibition of caspase-8 promotes RIP1/RIP3 complex formation, resulting in death signaling-induced necroptosis [19, 20]. However, this did not seem to be the case in these experiments, where inhibition of caspase-8 had less of an effect than did inhibition of caspase-2 or -9 (Fig 5B and 5D). Furthermore, the pan-caspase inhibitor Z-VAD had no effect on TRAIL-induced necroptosis of pancreatic cancer cells.

Caspase-2 has been referred to as an “orphan” caspase [25], and its role in TRAIL-induced apoptosis of cancer cells has not been established fully. It has been reported that caspase-2 is necessary for optimal TRAIL-mediated cleavage of Bid in human colon cancer cells [47]. In addition, caspase-2 primes cancer cells for TRAIL-induced apoptosis by processing procaspase-8 [48]. In this study, we showed that inhibition of caspase-2 suppressed apoptosis in TRAIL-treated cancer cells. Alternatively, ROS activate caspase-2, and DNA damage also induces its cleavage [49]. We recently reported that ROS trigger DNA damage, thereby leading to activation of caspase-2 in renal cell carcinoma lines [50]. Caspase-2 activation in response to DNA damage provides an important link between such damage and engagement of the apoptotic pathway [50, 51]. Additionally, ROS triggered caspase-2 activation and induced apoptosis in a human leukemic T cell line [51]. However, in this study, we found no crosstalk between ROS and caspases (Fig 6).

The expression of either DR4 or DR5 is prerequisite for TRAIL-induced apoptosis. The five cell lines studies were positive for DR5, but not for DR4 (Fig 1B). Therefore, the TRAIL-induced apoptosis of MiaPaCa-2 and BxPC-3 cells must act through DR5. By contrast, the other two pancreatic cancer cell lines and PrEC cells were relatively resistant to TRAIL, although they expressed DR5. Although DcRs are thought to prevent TRAIL-induced apoptosis in normal cells [4], normal epithelial PrEC cells partially expressed DcR2 to the same level as TRAIL-sensitive MiaPaCa-2 cells. Therefore, the expression of DcRs cannot account for TRAIL-resistance of Panc-1 and AsPC-1 cells, as well as that of PrEC cells. However, TRAIL-mediated apoptosis is regulated by several other mechanisms [52]: dysfunction or mutations in DR4/DR5; defects in the death-inducing signaling complex; a defect in effector caspases; and changes in pro-apoptotic or anti-apoptotic proteins. At this time, the differences in TRAIL-sensitivity among the five cell lines used in this study remain unclear.

In conclusion, we show that peroxide is involved in TRAIL-induced apoptosis in human pancreatic cancer MiaPaCa-2 cells, and that both peroxide and superoxide play regulatory roles in necroptosis of TRAIL-treated cells. These results show that TRAIL can induce two types of cell death, i.e., apoptosis and necroptosis, in human pancreatic cancer cells under specific conditions. Necroptosis can induce inflammation more vigorously than apoptosis [17, 18], but ROS play regulatory roles in cell death-associated inflammation via preferential inhibition of necroptosis compared to apoptosis. Dying cancer cells might produce ROS to inhibit cell death-associated inflammation. Given that pancreatic cancer is accompanied by inflammation [53], this study of necroptosis should increase our understanding of the pathophysiology of pancreatic cancer.

## Supporting Information

S1 Fig Autophagy in BxPC-3 cells treated with Tempol. (A) Four pancreatic cancer cell lines were examined for expression of LC3 by immunoblot. (B) LC3 expression in BxPC-3 cells treated with Tempol (1 mM) for 24 h was examined by immunoblot. β-Actin was used as the control.
(TIF)
