# Supplementary figures and images for: The Roles of ROS and Caspases in TRAIL-Induced Apoptosis and Necroptosis in Human Pancreatic Cancer Cells
Source: PLoS One. 2015 May 22;10(5):e0127386. doi: 10.1371/journal.pone.0127386 (PMC4441514; doi:10.1371/journal.pone.0127386)

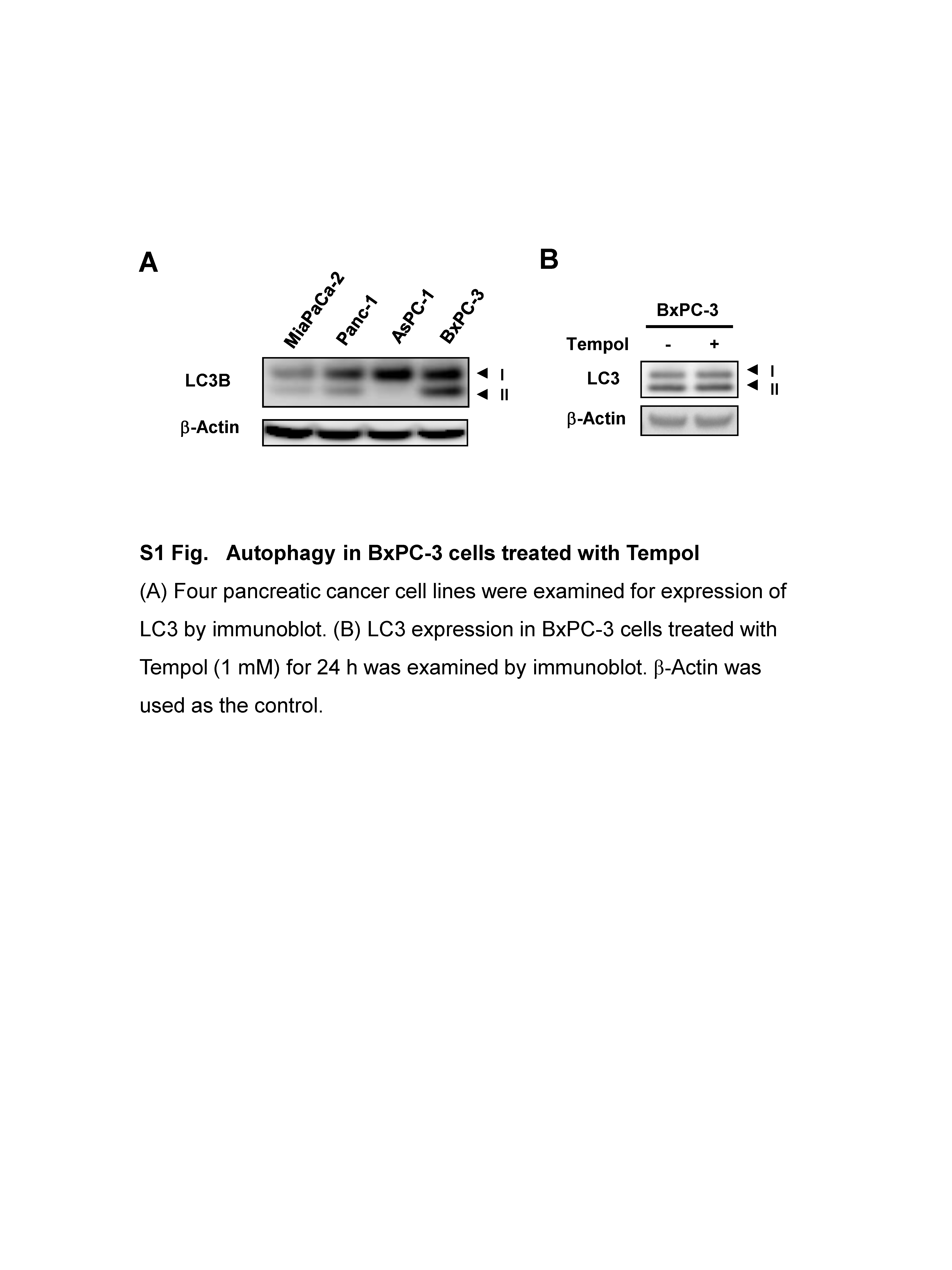

Supplement: S1 Fig — (A) Four pancreatic cancer cell lines were examined for expression of LC3 by immunoblot. (B) LC3 expression in BxPC-3 cells treated with Tempol (1 mM) for 24 h was examined by immunoblot. β-Actin was used as the control. (TIF) [file pone.0127386.s001.tif]
